# Targeting the Intestinal Microbiota: A Novel Direction in the Treatment of Inflammatory Bowel Disease

**DOI:** 10.3390/biomedicines12102340

**Published:** 2024-10-15

**Authors:** Jie Zhang, Huilin Gan, Xiaoyan Duan, Guangming Li

**Affiliations:** Department of Gastroenterology, Xinhua Hospital, School of Medicine, Shanghai Jiaotong University, Yangpu District, Shanghai 200092, China; zhangjie0912@sjtu.edu.cn (J.Z.); kamwailam@sjtu.edu.cn (H.G.)

**Keywords:** inflammatory bowel disease, intestinal microbiota, enteral nutrition, fecal microbiota transplantation, probiotics

## Abstract

Over the past decade, there has been a rapid increase in the incidence of inflammatory bowel disease. It has been suggested that multifactorial interactions of environmental factors, genetic factors, immune response and intestinal microbiota are involved in the pathogenesis of inflammatory bowel disease. It is widely recognized that the intestinal microbiota are essential for human metabolism, the immune system and pathogen resistance, and are integral to human health. Therefore, the dysbiosis of the microbiota is a critical step leading to intestinal mucosal damage and a key factor in the pathogenesis of inflammatory bowel disease. Regulating the microbiota through interventions such as enteral nutrition, fecal microbiota transplantation, and probiotic supplementation has the potential to prevent or even reverse intestinal dysbiosis, opening up new perspectives for the treatment of inflammatory bowel disease.

## 1. Introduction

Inflammatory bowel disease (IBD) encompasses Crohn’s disease (CD) and ulcerative colitis (UC), characterized by chronic inflammation of the intestinal mucosa and featuring recurring episodes of inflammation and remission [1]. Recently, there has been a global increase in the incidence and prevalence of IBD, especially among adolescents. Chronic inflammation associated with IBD is a risk factor for colorectal cancer, impacting prognosis significantly [2]. The pathogenesis of IBD involves genetic susceptibility, environmental factors and abnormal immune response. All of the above factors will directly or indirectly lead to the dysbiosis of the intestinal microbiota, which in turn triggers IBD [3].

With the breakthrough of new technologies, such as the advancements in next-generation sequencing technology, researchers have gained a deeper understanding of the intestinal microbiota. In the intestinal mucosa, the activation and differentiation of the host immune system plays a pivotal role. The intestinal microbiota guides the functional maturation of both the innate and adaptive immune systems, enabling the proper recognition and response to pathogens. Simultaneously, it regulates immune responses to prevent the excessive activation of the immune system, thus reducing inflammation and the occurrence of autoimmune diseases [4,5]. In a healthy human, the intestinal microbiota are predominantly composed of *Firmicutes* and *Bacteroidetes*, along with smaller proportions of *Proteobacteria* and *Actinobacteria*, which together account for more than 99% of the total gut bacterial population. Recent studies have shown that in patients with IBD, there is an increase in oxidative stress within the gut, leading to a decrease in the abundance of *Bacteroidetes*, *Firmicutes* and *Actinobacteria*, while *Proteobacteria* levels increase [6]. This dysbiosis results in reduced microbial diversity, the disruption of the intestinal mucosal barrier and subsequent gut inflammation [7,8]. Although fungi make up a relatively small fraction of the gut microbiome, recent research has begun to uncover their role in modulating intestinal inflammation. For example, *Candida albicans* has been found to promote pro-inflammatory responses, while *Saccharomyces boulardii* exerts anti-inflammatory effects [9]. Therefore, an abnormal microbial community structure and the dysbiosis of the host microbiota can significantly impact intestinal immune function and immune homeostasis. This imbalance increases the risk of inflammatory responses and disrupts the integrity of the gut barrier. The translocation of microbial populations and their components (such as toxins) will move into the circulation, consequently promoting intestinal inflammation. Conversely, intestinal inflammation can, in turn, impact the composition and functionality of the microbiota [10]. Consequently, intestinal microbiota dysbiosis has been widely recognized to play a key role in the pathogenesis of IBD. Modulating the intestinal microbiota provides crucial insights into IBD treatment, thereby propelling the development of novel therapeutic strategies.

## 2. The Intestinal Microbiota and IBD

### 2.1. Intestinal Microbiota

**Figure 1:**
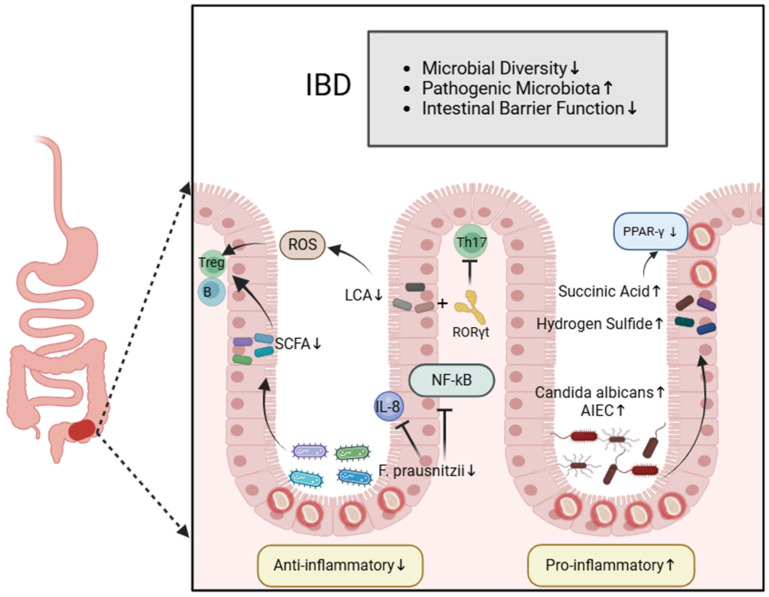
The dysbiosis of intestinal microbiota in inflammatory bowel disease. a. Weakened anti-inflammatory effects: (1) In patients with IBD, the abundance of *F. prausnitzii* is reduced, weakening its role in blocking the activation of the NF-kB pathway and reducing the production of the pro-inflammatory cytokine IL-8. (2) The reduction in intestinal microbiota metabolites like SCFAs in IBD patients weakens mucosal immune regulation, as SCFAs play a key role in promoting B cell development and the differentiation and expansion of Tregs. (3) LCA inhibits the differentiation of Th17 cells by directly binding to the key transcription factor RORγt. Conversely, LCA promotes the differentiation of Tregs by inducing mitochondrial reactive oxygen species (ROS) production. b. Enhanced pro-inflammatory effects: (1) Pathogenic bacteria, such as Adherent-invasive *Escherichia coli* (AIEC) and *Candida albicans*, increase in IBD patients, damaging epithelial integrity and invading the mucosal barrier. (2) Gut microbial metabolites such as succinic acid and sulfate-reducing bacteria (SRB) increase in IBD patients. Succinic acid downregulates the anti-inflammatory factor PPAR-γ, promoting inflammation progression. SRB produces toxins that damage intestinal epithelial integrity, exacerbating the inflammatory response.

The intestinal microbiota represents the largest reservoir of microorganisms within the human body, comprising thousands of diverse bacteria, fungi and viruses, continually attracting the attention of researchers. The microbiota and their metabolites collectively govern the functional equilibrium of the gastrointestinal environment, assisting in maintaining the biological barrier, nutrient metabolism and regulating immune responses. The intestinal microbiota forms a barrier through their close interactions with intestinal epithelial cells, preventing the entry of pathogens and harmful substances into the body. The metabolic products of the intestinal microbiota serve as key molecular mediators between the microbial community and the host, directly influencing the function of immune cells [11]. Among these metabolites, short-chain fatty acids (SCFAs), which are the primary by-products of dietary fiber fermentation by intestinal microbiota, play a crucial role. SCFAs not only provide an energy source for intestinal epithelial cells but also possess significant immunomodulatory functions. SCFAs can help to maintain immune balance by inhibiting the production of pro-inflammatory mediators and promoting the secretion of anti-inflammatory cytokines [12,13]. Furthermore, the intestinal microbiota have the ability to regulate both innate and adaptive immunity in the host, influencing a wide range of immune processes and contributing to overall immune homeostasis. First, the gut’s commensal bacteria play a crucial role in regulating the development of mucosal immunity, preventing the invasion of exogenous pathogens. Once pathogens breach the intestinal barrier, the gut swiftly activates innate and adaptive immune mechanisms to combat them. A key feature of intestinal innate immunity is the recognition of potential pathogens and harmless antigens via pattern recognition receptors (PRRs). Toll-like receptors (TLRs), as part of the PRR system, detect microbial-associated molecular patterns (MAMPs) from pathogenic microorganisms. Both commensal and pathogenic bacteria express MAMPs, but due to the mechanisms of immune tolerance, commensal bacteria do not trigger an immune response. Adaptive immunity predominantly occurs in gut-associated lymphoid tissues, where the intestinal microbiota interacts with adaptive immune cells such as B cells, T cells and natural killer (NK) cells, promoting the generation and function of regulatory T cells (Tregs) [14,15]. Cytokines are key signaling molecules in the immune system. The intestinal microbiota, under homeostatic conditions, interact with the host immune system to induce the production of pro-inflammatory and anti-inflammatory cytokines [16] (Table 1). Pro-inflammatory cytokines activate innate immune cells and enhance cytotoxic functions, thereby protecting the gut from pathogen invasion. In contrast, anti-inflammatory cytokines regulate the activation of Tregs, preventing excessive inflammatory responses in the gut [17]. The balance between pro-inflammatory and anti-inflammatory cytokines within the gut is central to maintaining intestinal homeostasis. Under normal conditions, the intestinal mucosa remains tolerant to the resident intestinal microbiota. However, when dysbiosis occurs, this tolerance is disrupted. The translocation of large quantities of bacteria and continuous antigenic stimulation lead to the activation of the intestinal mucosal immune system. This hyperactivation causes an imbalance between pro-inflammatory and anti-inflammatory responses, which disrupts immune homeostasis in the gut and directly contributes to the pathogenesis of IBD [18,19] (Figure 1).

### 2.2. Intestinal Microbiota Associated with Anti-Inflammatory Effects

The role of the intestinal microbiota in the development of inflammation has been validated through animal experiments. Research indicates that germ-free rodents do not develop IBD [26]. Inducing intestinal inflammation is possible by transferring inflammatory microbial communities from diseased mice to healthy ones. Consequently, a germ-free gut environment can prevent genetically susceptible mice from developing colitis [27]. Franzosa EA et al. discovered that, compared to healthy individuals, patients with IBD exhibit decreased diversity in their intestinal microbiota, notably characterized by decreased *Firmicutes* and increased *Proteobacteria* [28].

Specifically, in patients with CD, the decreased abundance of *Roseburia intestinalis* and *Faecalibacterium prausnitzii* has been detected, while the abundance of *Ruminococcus gnavus* has increased [28]. In a study by Shen Z et al., it was found that when human CD4 T cells were co-cultured with dendritic cells, *Roseburia intestinalis* was able to induce dendritic cells activation via interactions with intestinal epithelial cells, promoting the differentiation of Tregs and suppressing inflammation [29]. Additionally, Henke M et al. discovered that in mice, *Ruminococcus gnavus* could induce dendritic cells to secrete the pro-inflammatory cytokine TNF-α, contributing to an inflammatory response [30]. Among these microbial groups, the reduced levels of *Faecalibacterium prausnitzii (F. prausnitzii)* have received particular attention in IBD research. Reports indicate that *F. prausnitzii* can inhibit NF-kB activation and IL-8 production, demonstrating anti-inflammatory properties in the gut. Furthermore, it synthesizes a non-conventional protein known as the microbial anti-inflammatory molecule (MAM), which inhibits the NF-kB pathway in epithelial cells, thereby exhibiting anti-inflammatory activities [31]. Consequently, the decreased presence of this anti-inflammatory *Firmicute* may lead to the further progression of intestinal inflammation. Observing low levels of *F. prausnitzii* in fecal samples can forecast relapse in patients with IBD during remission, highlighting significant clinical significance [32]. Additionally, researchers have found that the abundance of *Saccharomyces* spp. is reduced in patients with IBD. *Saccharomyces* spp. exert anti-inflammatory effects by modulating T cells migration and infiltration, as well as reducing the release of pro-inflammatory cytokines in inflamed mucosa. For instance, *Saccharomyces boulardii* can alleviate local intestinal inflammation by limiting the migration of dendritic cells to inflamed sites. It also induces the secretion of IL-8, IFN-γ, and TGF-β, which contribute to the anti-inflammatory response in IBD patients [33,34].

The progression of metabolomics technology has aided in the identification of alterations to metabolites like SCFAs, bile acids, and amino acid levels within fecal samples of IBD patients [35]. SCFAs like butyric acid, propionic acid, and acetate salts provide abundant energy for the intestinal epithelium. They regulate mucosal immunity by promoting B cell development, regulating the differentiation and expansion of Tregs, and inhibiting the IL-6 and IL-17 pathways, enhancing the anti-inflammatory effects of Foxp3. Additionally, butyric acid can act upon immune cells in the intestinal mucosa, potentially increasing the number and activity of Tregs. We found that such metabolites are reduced and may contribute to exacerbating inflammation in IBD patients. Bile acids produced by the intestinal microbiota also play a crucial role in T cell differentiation. A study identified two different lithocholic acid (LCA) derivatives—3-oxoLCA and isoalloLCA—with distinct regulatory effects on mouse T cells. 3-oxoLCA inhibits the differentiation of Th17 cells by directly binding to the key transcription factor RORγt. Conversely, isoalloLCA promotes the differentiation of Tregs by inducing mitochondrial reactive oxygen species (ROS) production [36]. However, alterations in the intestinal epithelium induced by IBD could potentially hinder the reabsorption of bile acids, thereby dampening their regulatory influence on T cells, and this further exacerbates inflammation. These findings underscore the significant role of the microbiome and its metabolites in the development of intestinal inflammatory diseases.

### 2.3. Intestinal Microbiota Associated with Pro-Inflammatory Effects

When the intestinal microbiota are dysregulated, pathogenic bacteria proliferate extensively and colonize the intestinal tract, disrupting the intestinal mucosal barrier and triggering inflammation within the body. For instance, in IBD, there is an increased abundance of Adherent-invasive *Escherichia coli* (AIEC). These bacteria can adhere to and invade intestinal epithelial cells, colonize the intestinal mucosa and stimulate the release of pro-inflammatory factors [37]. Jean-Félix et al. discovered that pathogens such as *Clostridium difficile*, *Escherichia coli*, *Listeria monocytogenes* and *Salmonella typhimurium* can adhere to intestinal mucins, disrupt epithelial cells and invade the mucosa at ulcer sites, leading to the promotion of inflammation [38]. Li et al. analyzed the fungal profiles of both the intestinal mucosa and fecal samples from patients with active CD and healthy individuals. They discovered that, compared to non-inflamed mucosa, inflamed mucosa exhibited an increase in fungal richness and diversity, characterized by a significant increase in species such as *Candida* spp., *Gibberella moniliformis*, *Alternaria brassicicola* and *Cryptococcus neoformans* [39]. Similarly, fungal dysbiosis was observed in patients with UC. Using sequencing and culture techniques, researchers compared the colonic mucosa of UC patients and healthy controls. The findings revealed a marked increase in *Candida albicans* in the gut mucosa of UC patients. Further investigation identified that *candidalysin*, a toxin secreted by *Candida albicans*, is a key factor in inducing mucosal immune responses and promoting intestinal inflammation [40]. These pathogenic bacteria intensify the severity of intestinal inflammatory diseases.

Additionally, succinic acid metabolism has emerged as a novel area of research in IBD. Succinic acid is a tricarboxylic acid cycle intermediate, produced by host cells and the microbial community A140141. In the host, succinate acts as a crucial pro-inflammatory signal, leading to the downregulation of the anti-inflammatory factor PPAR-α [41]. Studies by Ehud Ohana et al. confirmed a significant increase in succinic acid levels within the intestines of IBD patients [42], showing that it exhibits pro-inflammatory effects. Another example of altered functionality within the intestinal microbiota in UC is the increased presence of sulfate-reducing bacteria (SRB). Hydrogen sulfide, a byproduct of SRB metabolism, can exert adverse effects on the intestinal environment and microbiota by its high concentration, causing toxicity and lowering pH levels, consequently inducing mucosal inflammation [43,44].

These research findings highlight the significant relevance of the composition and functionality of the intestinal microbiota in the development and severity of IBD. So, we created a table that briefly illustrates the role of the microbiota in IBD (Table 2). Therefore, understanding and modulating the composition and metabolic activities of the microbiota holds substantial significance in preventing and treating IBD.

## 3. Therapies Targeting the Intestinal Microbiota

Current treatment strategies for IBD primarily focus on controlling inflammation through medication, including corticosteroids, aminosalicylates, immunomodulators and biologics [48]. Corticosteroids (e.g., prednisone) and aminosalicylates (e.g., 5-aminosalicylic acid) help to alleviate inflammation by inhibiting the release of pro-inflammatory mediators such as prostaglandins and leukotrienes [49,50]. Immunomodulators (e.g., azathioprine) suppress the proliferation of inflammatory cells and are mainly used in clinical practice to induce or maintain remission, particularly in steroid-dependent CD patients who are difficult to manage. Despite their efficacy in relieving IBD symptoms, aminosalicylates, corticosteroids and immunomodulators have limitations, including high relapse rates after discontinuation and low patient adherence with long-term use. Biologics, now widely used in clinical practice, acts primarily on the immune system with different targets. For example, TNF inhibitors antagonize TNF-α, neutralizing its biological activity and reducing inflammation. Integrin inhibitors, on the other hand, block the binding of α4β7 integrin to its ligand, preventing lymphocyte migration to inflamed intestinal mucosa and inhibiting localized immune responses [51]. While biotherapy is effective for many patients, up to 30% of individuals show no response to initial treatment, and up to 50% experience diminishing responses over time [5]. Patients with IBD are at high risk for opportunistic infections. Research has shown that the use of corticosteroids, immunomodulators and biologics can significantly suppress the immune system in IBD patients, leading to an increased risk of opportunistic infections and a higher incidence of malignancies [52]. As a result, current medications often fall short in addressing the fundamental causes of IBD, such as the dysfunction of intestinal mucosal barriers and the dysbiosis of intestinal microbiota [53], which could result in severe adverse reactions. In certain circumstances, many patients may ultimately require surgical intervention.

**Figure 2:**
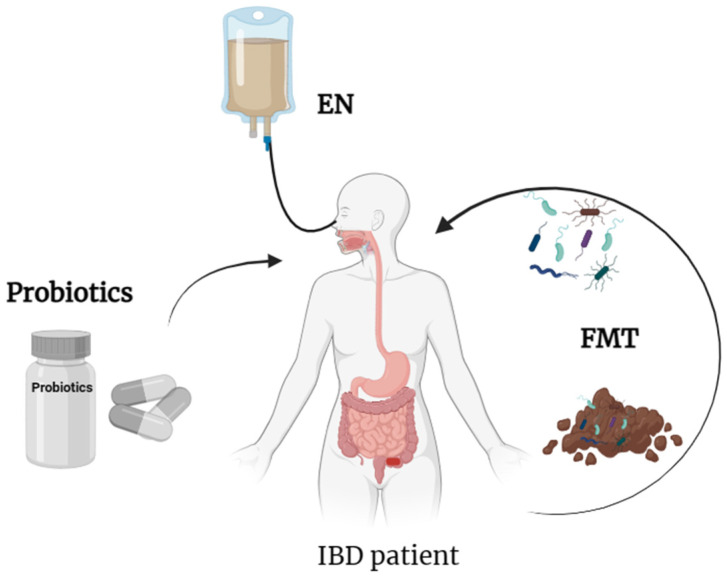
Therapies targeting the intestinal microbiota. (1) EN provides nutritional formulas to patients with IBD through oral ingestion or nasogastric tube delivery. (2) FMT involves introducing fecal microbiota from healthy individuals into the patient’s gastrointestinal tract. (3) Oral probiotics induce positive changes in the composition of the intestinal microbiota.

With the inception and development of the dysbiosis theory regarding the intestinal microbiota, the research on targeting the microbiota in IBD treatment has exponentially intensified. This therapeutic approach aims to restore intestinal health, alleviate inflammation and improve symptoms by modulating the composition and functionality of the microbiota. This may involve interventions such as enteral nutrition (EN), or the use of probiotics or fecal microbiota transplantation (FMT), among others, to regulate the intestinal microbial community (Figure 2). These innovative treatment strategies could prove beneficial for patients with poor responses to traditional medication therapies or who experience severe adverse reactions.

### 3.1. Enteral Nutrition

Enteral nutrition therapy is recommended for patients with IBD who are malnourished or at nutritional risk, according to a consensus of experts in nutritional support therapy for IBD [54]. EN is a specialized diet in which a liquid nutritional formula is delivered to the body via oral or nasogastric tube, and the course of treatment is usually 6–8 weeks [55,56]. It not only provides essential nutrients, but also conforms to the physiologic digestive absorption pathways, maintaining the integrity of the intestinal mucosal barrier. Its effectiveness is comparable to corticosteroids, yet it tends to cause fewer adverse effects.

In pediatric and adolescent patients with CD, EN stands as the primary preferred treatment for initiating remission [57]. The exact mechanisms through which EN operates are not entirely understood. However, it is hypothesized that EN may decrease the production of inflammatory molecules in the intestinal lining and rectify imbalances in the intestinal microbiota. In addition, it causes a positive change in the microbiota from an initial pro-inflammatory state to an anti-inflammatory state. These actions potentially lead to an improvement in intestinal inflammation and facilitate the healing of the intestinal lining [58]. Studies have shown that EN therapy leads to positive changes in the intestinal microbiota, shifting from an initially pro-inflammatory state to an anti-inflammatory one, thereby promoting disease remission. In individuals with CD undergoing EN treatment, imbalances in the intestinal microbiota primarily involve a notable decrease in the *Bacteroidetes* and *Firmicutes phyla*, coupled with an increase in *Enterobacteriaceae*. In a prospective clinical study of pediatric CD patients, changes in fecal bacterial communities were reported by 16S rRNA sequencing after 2 weeks of EN, showing an increase in *Bacteroidetes* and the restoration of microbial balance [59].

Interestingly, Gerasimidis et al. discovered that in patients experiencing remission due to EN treatment, there was an increase in sulfur components and a decrease in butyrate salts in their feces, leading to an overall reduction in bacterial diversity. This unusual ecological environment in the intestine correlates with the healing of the intestinal lining and the reduction in clinical disease symptoms, which contradicts findings from earlier research [60].

Upcoming research endeavors are directed towards investigating the mechanisms by which EN influences the microbiota and aims to devise precise strategies to manage changes in the intestinal microenvironment.

### 3.2. Fecal Microbiota Transplantation

FMT is a therapeutic approach that involves introducing the fecal microbiota obtained from healthy donors into the gastrointestinal tract of patients to restore the abnormal microbial composition within the intestines [61]. Donor feces can be administered to patients through various methods, including a colonoscopy, enemas, upper gastrointestinal tract delivery, or oral ingestion via frozen capsules. Studies have confirmed the comparable effectiveness of these aforementioned administration methods [62]. The success of FMT lies in the ability to reestablish and restore the abnormal microbial community within the patient’s intestines to an ideal healthy state.

In 1989, FMT was first used in patients with UC. Long-term follow-up revealed that the initially treated patient maintained a disease-free status for over 20 years [63]. A meta-analysis incorporating 23 cohort studies involving 319 patients with varying disease severity who received FMT treatment found that 93 patients achieved clinical remission. The clinical remission rate was 20% for mild IBD and 30% for moderate to severe IBD. The study suggested that patients with moderate to severe IBD might benefit more from FMT than those with a mild form of the disease [64]. Imdad A et al. also summarized four large, randomized studies, demonstrating a significant increase in the clinical remission rate for IBD patients following FMT. The remission rate doubled compared to the control group [65].

It is interesting to note that the efficacy of FMT in treating IBD currently presents a mixed scenario. A prospective study by Vaughn et al. assessed clinical responses to FMT in 19 patients with CD. The results showed that 58% of patients experienced clinical remission. The analysis of samples before and after FMT in 15 of the subjects revealed a significant increase in the diversity of the intestinal microbiota post-FMT. However, the study also reported adverse events. One patient developed urticaria and another experienced worsening clinical symptoms. The latter patient had multiple underlying chronic conditions and ultimately required surgical intervention [66]. In summary, FMT demonstrates relatively high safety in IBD. Most commonly reported adverse events include abdominal pain, bloating, nausea, upper respiratory tract infections, headache, dizziness and fever [67]. The occurrence rate of severe adverse events such as the exacerbation of ulcerative colitis, infections, small bowel perforation and pneumonia is low.

However, current research related to FMT in IBD mostly consists of small-scale studies primarily reliant on clinical observations, thus lacking objectivity and reliability due to the influence of multiple factors. Furthermore, there is currently no standardized protocol for donor selection, stool preparation methods or dosing frequency in FMT, rendering it within the realm of clinical trials. The occurrence rate of severe adverse events remains uncertain, as does its efficacy, warranting further multicenter, high-quality randomized controlled trials to confirm its effectiveness and safety.

### 3.3. Probiotics

Probiotics are beneficial microorganisms within the intestinal that have been widely used in the treatment of various diseases, including inflammation, tumors, obesity, diabetes and others [68,69,70,71]. Firstly, they exert direct antimicrobial effects in the human body by producing substances such as bacteriocins, hydrogen peroxide and defensins. Secondly, they enhance intestinal barrier function by increasing mucus production and promoting the formation of tight junctions, which in turn reduces intestinal mucosal permeability [72]. Additionally, they regulate immune responses by modulating immunoglobulin production and the generation of pro-inflammatory cytokines. Through the regulation of the NF-κB pathway, they decrease the production of pro-inflammatory cytokines (e.g., IL-8, TNF-α, IFN-γ) and induce the production of anti-inflammatory cytokines, such as IL-10 and TGF-β [73]. In recent years, probiotics have shown potential as a therapeutic strategy in clinical trials for IBD (Table 3). For instance, Ballini et al. conducted a study where 40 IBD patients were randomly assigned to receive either probiotics or a placebo for 90 days. The results showed that oral probiotics effectively and safely regulated oxidative stress levels and alleviated gut inflammation in IBD patients [74].

Currently, the most commonly used probiotics include *Lactococcus, Lactobacillus, Bifidobacterium* and *Escherichia coli* Nissle 1917. *Lactobacillus plantarum* can reduce the production of pro-inflammatory cytokines and prevent pathogens from adhering to the intestinal epithelial layer. *Bifidobacterium* helps to prevent barrier disruption and promotes the repair of damaged cells, maintaining the stability of the intestinal epithelial barrier. Specifically, the *Bifidobacterium bifidum BGN4-SK* strain improves DSS-induced colitis by producing antioxidant enzymes, increasing the expression of tight junction genes and reducing pro-inflammatory cytokines such as IL-6, IL-1β and TNF-α [19]. *Escherichia coli* Nissle 1917 is a well-known immunomodulator capable of stimulating the increase in anti-inflammatory cytokines, such as IL-10, and triggering an IgA-mediated immune response. Lactobacillus plantarum, on the other hand, can reduce the production of pro-inflammatory cytokines and prevent pathogens from adhering to the intestinal epithelium [75]. VSL#3 is a high-concentration blend composed of eight microbial strains and is considered a promising probiotic [76]. Sood et al. conducted a multicenter, randomized, double-blind, placebo-controlled trial that demonstrated the beneficial effects of VSL#3. Adult patients with mild to moderately active UC received VSL#3 supplementation twice daily for 12 weeks. The treatment outcomes were measured using the ulcerative colitis disease activity index (UCDAI) score. The study results revealed that patients in the VSL#3 group showed significantly decreased UCDAI scores and enhanced mucosal healing compared to the placebo group [77]. Researchers have confirmed that VSL#3 regulates the host’s immune response and improves epithelial barrier function by increasing the anti-inflammatory cytokine IL-10 and suppressing the secretion of pro-inflammatory cytokines such as TNF-α and IFN-γ [78]. Interestingly, VSL#3 has been shown to have a synergistic effect when used in conjunction with conventional medications. For instance, when combined with 5-aminosalicylic acid, it can enhance anti-inflammatory effects and inhibit leukotrienes and IL-1 production. Additionally, when used in combination with balsalazide, it is more effective in relieving UC compared to balsalazide alone [73]. Moreover, VSL#3 can also prevent pouchitis, a postoperative complication in UC patients [79]. Overall, long-term treatment with VSL#3 in patients with IBD is likely to lead to a better prognosis.

Therefore, probiotics play a crucial role in regulating the intestinal microbiota and intestinal immunity, helping to address the shortcomings of conventional drug therapies. By modulating the immune response, maintaining intestinal barrier integrity, and reducing inflammation, probiotics can complement traditional treatments, offering a more holistic approach to managing gut-related disorders such as IBD. However, the clinical response of patients to probiotics is influenced by various potential interrelated factors such as host genetics, lifestyle, dietary habits, and the composition of the endogenous microbiota [80]. Moreover, maintaining the viability of probiotics poses a significant technical challenge. Many live strains may die during storage and transportation, leading to their ineffectiveness before patients can derive any health benefits. We look forward to future research exploring the identification and combination of various probiotic strains to uncover their synergistic effects. This will enable the development of personalized and individualized therapies tailored to specific IBD phenotypes. By optimizing the selection of probiotic strains for each patient’s unique microbiome composition and immune response, it is possible to enhance therapeutic outcomes, complementing traditional treatments and providing more precise, targeted approaches for managing IBD.

## 4. Conclusions

Based on the above, we believe that we can try to utilize the characterization of the intestinal microbiota as a therapeutic and prognostic biomarker for IBD. For instance, some studies indicate an increase in *Firmicutes* among UC patients responding to mesalazine [81]. Additionally, CD patients with an intestinal microbiota composition similar to healthy controls have lower relapse rates compared to those with dysbiosis [82]. Based on these findings, future medical advancements may focus on utilizing the intestinal microbiota’s features for individualized therapy. This may involve employing targeted therapies to protect the microbiota, correct microbial metabolic activities and restore the immune system regulation, thus effectively and safely managing IBD patients. This personalized treatment approach holds promise as a significant innovation in future medical fields, providing better therapeutic outcomes for IBD patients. However, this field requires further research to validate and refine these concepts, translating them into viable treatment strategies for clinical practice.

## Figures and Tables

**Table 1:** Pro-inflammatory and anti-inflammatory cytokines.

Cytokines	Sources of Cytokines	Function	Impact on Inflammation	References
IL-1β	Neutrophils Macrophages	Disrupting the intestinal tight junction barrier and allowing increased intestinal penetration of luminal antigens.	Pro-inflammatory	Kaminsky L et al. [20]
IL-6	Macrophages DCs T cells	Increasing the intestinal tight junction permeability by stimulating the expression of channel-forming claudin-2 and undermining the integrity of the intestinal barrier.	Pro-inflammatory	Guo Y et al. [21]
TNF-α	Macrophages DCs Adipocytes	Inhibiting epithelial cell proliferation through the suppression of β-catenin/T cell factor signaling.	Pro-inflammatory	Okumura R et al. [22]
IL-10	Macrophages T cells	Activating the Janus kinases/signal transducer and transcription proteins pathway exerts its anti-inflammatory cytokine function while inhibiting the activation of NF-κB and its subsequent pro-inflammatory effects.	Anti-inflammatory	Papoutsopoulou S et al. [23]
IL-22	T cells	Inducing the expression of mucin genes in mucosal epithelial cells through STAT3-dependent signaling and enhancing intestinal barrier.	Anti-inflammatory	Keir M et al. [24]
TGF-β	Macrophages Mastocyte B cells	Enhancing the intestinal epithelial barrier function by inducing the production of the tight junction protein Claudin-1, and by preventing the pathogenic bacteria-induced reduction in levels of the tight-junction proteins Claudin-2.	Anti-inflammatory	Ihara S et al. [25]

**Table 2:** Changes in intestinal microbiota in IBD.

Microbiota	Functions	Abundance	Study Subjects	Research Context	Impact on IBD	Reference
*Roseburia intestinalis*	Intestinal epithelial cells induce dendritic cell activation, promoting helper T cell differentiation to suppress inflammation	Decrease	TLR-5-deficient mice; Bone marrow chimera mice	Determination of Treg differentiation and analysis of the correlation between TLR5/TSLP/TGFβ expression and *Roseburia intestinalis* by co-culture of human CD4 T cells with DCs	Decreased histopathological score	Shen Z et al. [29]
*Faecalibacterium*	Producing butyrate, inhibiting HDAC1, promoting Foxp3 and blocking the IL-6/STAT3/IL-17 pathway to exert anti-inflammatory effects	Decrease	Sprague Dawley rats; C57BL/6J mice	Study of metabolites of *Faecalibacterium prausnitzii* in IBD by gas chromatography-mass spectrometer	Mild mucosal inflammation with a relatively low level of neutrophil infiltration and mild edema	Zhou L et al. [45]
*Bifidobacterium*	Downregulating inflammatory cytokines and inhibiting the NF-κB pathway to regulate the intestinal immune system and protect intestinal epithelial cells	Decrease	Male Balb/c mice	Exploring the protective role of *Bifidobacterium bifidum* against DSS-induced colitis in mice by fecal microbiota	Alleviation of crypt distortion, diarrhea, blood in the stool, reduction in colon length and neutrophil infiltration	Yao S et al. [46]
*Escherichia coli*	Exhibiting survival and replication capability within macrophages and inducing the production of the inflammatory cytokine TNF-α	Increase	Patient-derived strains	Exploring *Escherichia coli* adhesion and invasion capacity in IBD by ribotyping	Prone to early recurrence	Lu Q et al. [5]
*Fusobacterium*	Expressing the FadA protein which leads to the adhesion and invasion of epithelial cells, disrupting the integrity of the intestinal mucosal barrier and stimulating the production of inflammatory cytokines IL-6, IL-8 and TNF-α	Increase	C57BL/6 male mice	Pathogenicity of *Fusobacterium* on mice with DSS-induced colitis analyzed by transmission electron microscope and transepithelial electrical resistance	Exacerbation of colitis as evidenced by severe crypt destruction, mucosal erosion and infiltration of numerous inflammatory cells	Liu H et al. [47]
*Ruminococcus*	Inducing dendritic cells to secrete the inflammatory cytokine TNF-α	Increase	C57BL/6 mice	Levels of the inflammatory cytokine TNFα were measured by enzyme-linked immunosorbent assay	Exacerbation of CD abdominal symptoms, even involving extraintestinal manifestations	Henke M et al. [30]

**Table 3:** Clinical trials on probiotics and IBD.

NCT Number	Title	Status	Conditions	Interventions	Primary Outcome Measures
NCT06392061	Effect of Probiotic Administration on Patients With Inflammatory Bowel Disease	Recruiting	Inflammatory bowel diseases	Dietary supplement: trilac	Disease course; Change in frequency of flares over 2 months; Change in hospitalizations rates over 2 months; Nutrition status
NCT05906043	A Multidisciplinary Approach to Assessing and Treating Fatigue in Inflammatory Bowel Disease	Recruiting	Inflammatory bowel diseases	Dietary supplement: probiotic Behavioral: exercise intervention Behavioral: acceptance and commitment therapy	Multidimensional fatigue index
NCT05666960	R-3750 in Patients With Mild to Moderate Ulcerative Colitis	Recruiting	Ulcerative colitis; Chronic mildulcerative colitis; Chronic moderate	Drug: R-3750	The number of participants with treatment-related adverse events after taking R-3750
NCT05652621	Efficacy of Probiotics in Patients With IBD	Recruiting	Ulcerative colitis	Dietary supplement: probiotics	Changes in intestinal microbiota
NCT04842149	The Effects of Bifidobacterium Breve Bif195 for Small Intestinal Crohn’s Disease	Active, not recruiting	Crohn’s disease	Dietary supplement: Bif195 capsules Dietary supplement: placebo capsules	Change in bowel wall thickness from baseline measured by intestinal ultrasound
NCT03266484	Effect of a Probiotic Mixture on the Gut Microbiome and Fatigue in Patients With Quiescent Inflammatory Bowel Disease	Active, not recruiting	Inflammatory bowel diseases	Dietary supplement: probiotic mixture Dietary supplement: placebo	Change in intestinal microbiota; Change in serum inflammatory cytokines levels; Change in metabolomic profiles; Change in fatigue symptoms
NCT04223479	Effect of Probiotic Supplementation on the Immune System in Patients With Ulcerative Colitis in Amman, Jordan	Completed	Ulcerative colitis	Drug: probiotic formula capsule Drug: placebos	Immunoglobulin G, immunoglobulin M, immunoglobulin A, IL-6, CRP, IL-1, IL-10, IL-12, TNF-α and complete blood count

## Abbreviations

Inflammatory bowel disease: IBD; Crohn’s disease: CD; ulcerative colitis: UC; *Faecalibacterium prausnitzii*: *F. prausnitzii*; microbial anti-inflammatory molecule: MAM; Short-chain fatty acids: SCFAs; regulatory T cells: Treg; lithocholic acid: LCA; reactive oxygen species: ROS; adherent-invasive *Escherichia coli*: AIEC; sulfate-reducing bacteria: SRB; enteral nutrition: EN; fecal microbiota transplantation: FMT; ulcerative colitis disease activity index: UCDAI; pattern recognition receptors: PRRs; Toll-like receptors: TLRs; microbial-associated molecular patterns: MAMPs.
